# Fluorescent and Bioluminescent Calcium Indicators with Tuneable Colors and Affinities

**DOI:** 10.1021/jacs.2c01465

**Published:** 2022-04-05

**Authors:** Nicole Mertes, Marvin Busch, Magnus-Carsten Huppertz, Christina Nicole Hacker, Jonas Wilhelm, Clara-Marie Gürth, Stefanie Kühn, Julien Hiblot, Birgit Koch, Kai Johnsson

**Affiliations:** † Department of Chemical Biology, Max Planck Institute for Medical Research, Jahnstrasse 29, 69120 Heidelberg, Germany; ‡ Department of Optical Nanoscopy, Max Planck Institute for Medical Research, Jahnstrasse 29, 69120 Heidelberg, Germany; § Institute of Chemical Sciences and Engineering, École Polytechnique Fédérale de Lausanne (EPFL), 1015 Lausanne, Switzerland

## Abstract

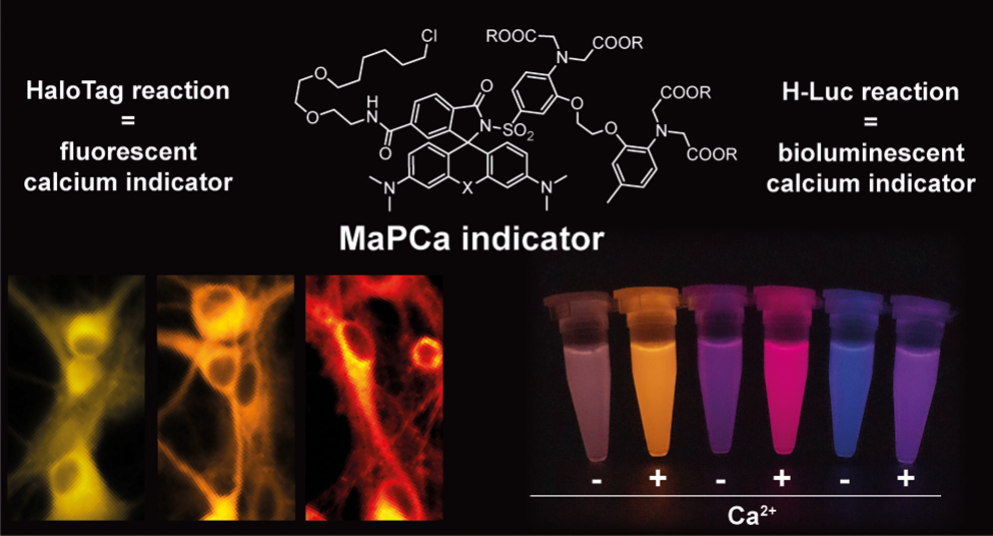

We introduce a family of bright, rhodamine-based calcium indicators with tuneable affinities and colors. The indicators can be specifically localized to different cellular compartments and are compatible with both fluorescence and bioluminescence readouts through conjugation to HaloTag fusion proteins. Importantly, their increase in fluorescence upon localization enables no-wash live-cell imaging, which greatly facilitates their use in biological assays. Applications as fluorescent indicators in rat hippocampal neurons include the detection of single action potentials and of calcium fluxes in the endoplasmic reticulum. Applications as bioluminescent indicators include the recording of the pharmacological modulation of nuclear calcium in high-throughput compatible assays. The versatility and remarkable ease of use of these indicators make them powerful tools for bioimaging and bioassays.

## Introduction

The second messenger calcium is involved in a plethora of signaling pathways and biochemical processes.^1^ The elucidation of its function in cellular processes has become possible largely through the development of calcium indicators.^2−4^ Although early development focused on synthetic calcium indicators, genetically encoded calcium indicators (GECIs) have now become the gold standard. The main reason for this is that GECIs can be genetically targeted to specific cellular populations and subcellular localizations, whereas the cellular uptake of synthetic calcium indicators lacks selectivity and is often inefficient. However, GECIs possess lower brightness, slower response kinetics, and a limited color range (especially in the far-red) in comparison to synthetic indicators.^5,6^ These limitations are of particular concern when highly localized areas, such as micro- and even nanodomains are investigated and more demanding microscopy techniques are used.^7−9^ A possibility to combine the brightness, response kinetics, and spectral range of synthetic fluorescent indicators with the targetability of GECIs is the use of self-labeling protein tags such as SNAP-tag and HaloTag.^10,11^ Self-labeling proteins form a covalent bond to a specific substrate and through this enable precise localization of synthetic molecules to proteins of interest. This approach has been used to create a number of localizable synthetic calcium indicators, for example, BG3-Indo-1,^12^ BOCA-1-BG,^13^ or RhoCa-Halo,^14^ and the far-red indicator JF_646_-BAPTA.^5,15^ However, these probes have limited cell permeability and solubility, and furthermore, require washing steps to remove unreacted probes, greatly limiting their applicability.^13,14^ The use of bright synthetic fluorophores for calcium sensing was enabled developing chemogenetic sensors in which the protein-based calcium-sensing domain calmodulin (CaM) interacts with an environmentally sensitive dye (*e.g.,* rHCaMP or HaloCaMP).^16,17^ However, based on the same calcium-sensing domain as most GECIs are, they suffer from relatively slow response kinetics.^16^ Furthermore, there is currently no localizable synthetic far-red calcium indicator with a suitable calcium affinity for calcium-rich areas such as the endoplasmic reticulum (ER) or calcium microdomains.^18,19^ Here, we present MaPCa dyes, a family of highly permeable calcium indicators with different colors and calcium affinities that can be coupled to HaloTag. As the reaction with HaloTag shifts the fluorescent scaffold of the indicator from a non-fluorescent into a fluorescent configuration, these probes can be used without any washing steps to remove the unbound probe.

## Results and Discussion

### Design Principle and Synthesis of MaPCa Dyes

**Figure 1:**
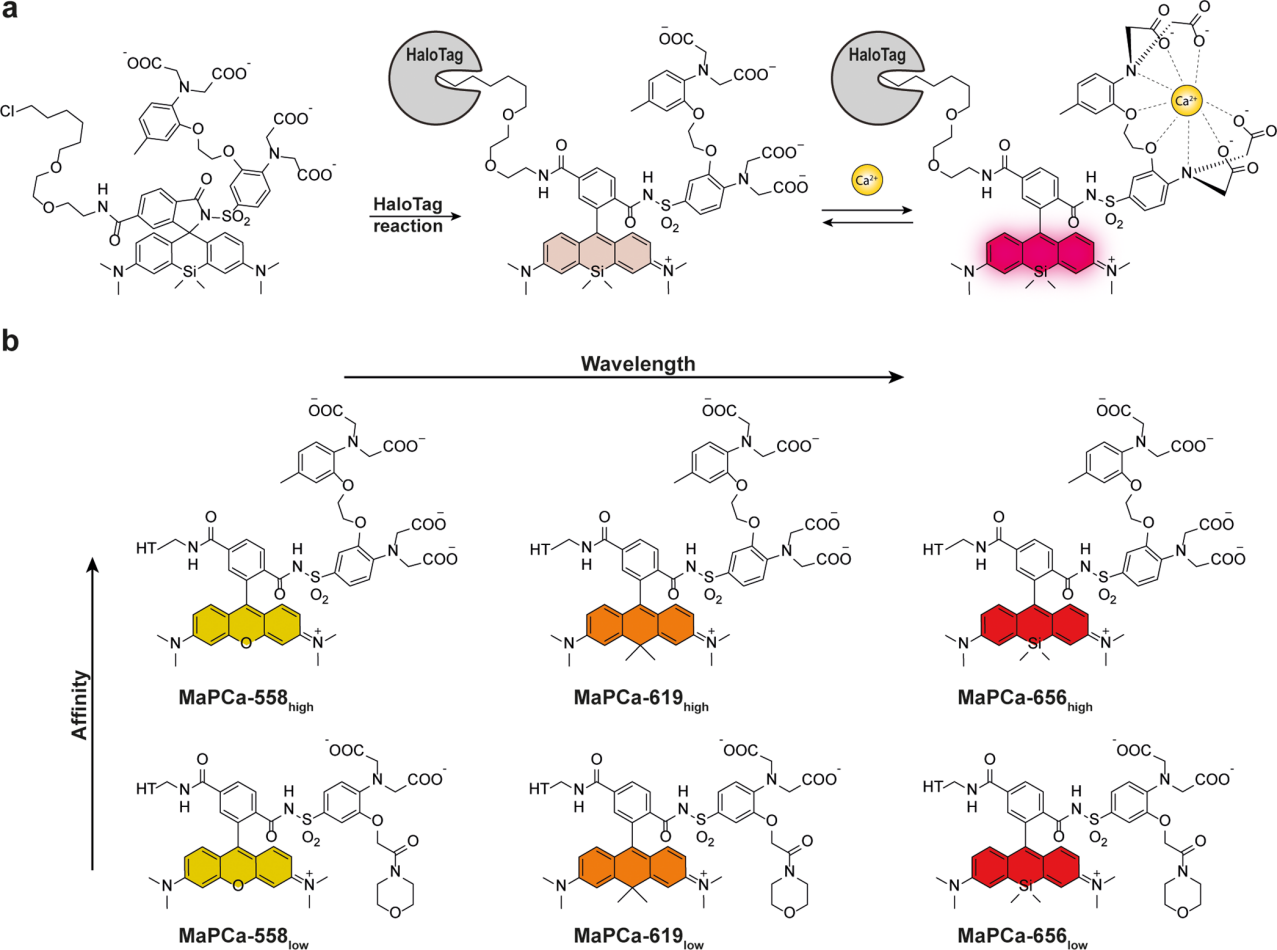
Schematic representation of the MaPCa dyes. (a) Representation of the double-turn-on mechanism of MaPCa dyes. Example for MaPCa-656_high_. If not bound to the HaloTag, MaPCa dyes are in their colorless, spirocyclic form. Upon binding to HaloTag, they open to their zwitterionic form and hence become potentially fluorescent, but PET-quenched by the Ca^2+^-binding moiety. Only upon calcium binding full fluorescence is achieved. (b) Overview of synthesized MaPCa dyes. HT = HaloTag-bound linker.

The design of our calcium indicators is based on the recently introduced MaP dyes, in which the lactone-forming carboxylic acid of a rhodamine is replaced with an amide attached to an electron-withdrawing group (*e.g.,* sulfonamides).^20,21^ This results in dyes that preferentially exist as a non-fluorescent spirolactam in solution, but shift to an open, fluorescent state upon binding to HaloTag, enabling no-wash imaging with a low background. We envisioned designing fluorogenic calcium indicators by attaching a calcium chelator such as BAPTA [1,2-bis(*o*-aminophenoxy)ethane-*N*,*N*,*N*′,*N*′-tetraacetic acid] through a benzene sulfonamide to the *ortho*-carboxylate of rhodamines and a chloroalkane (CA) through a carboxylate at the 6-position of the benzyl ring (Figure 1a). BAPTA would be thereby positioned in close proximity to the rhodamine core, which is an important factor for effective PET-quenching of the rhodamine by the free chelator.^15,22^ Attachment of the CA *via* the 6-position of the benzyl ring would enable HaloTag to shift the equilibrium from spirocyclization to the fluorescent, open form, thereby resulting in fluorogenicity. Furthermore, attachment of the CA *via* the 6-position would ensure a high labeling speed of the resulting HaloTag substrate.^23^

**Figure 2:**
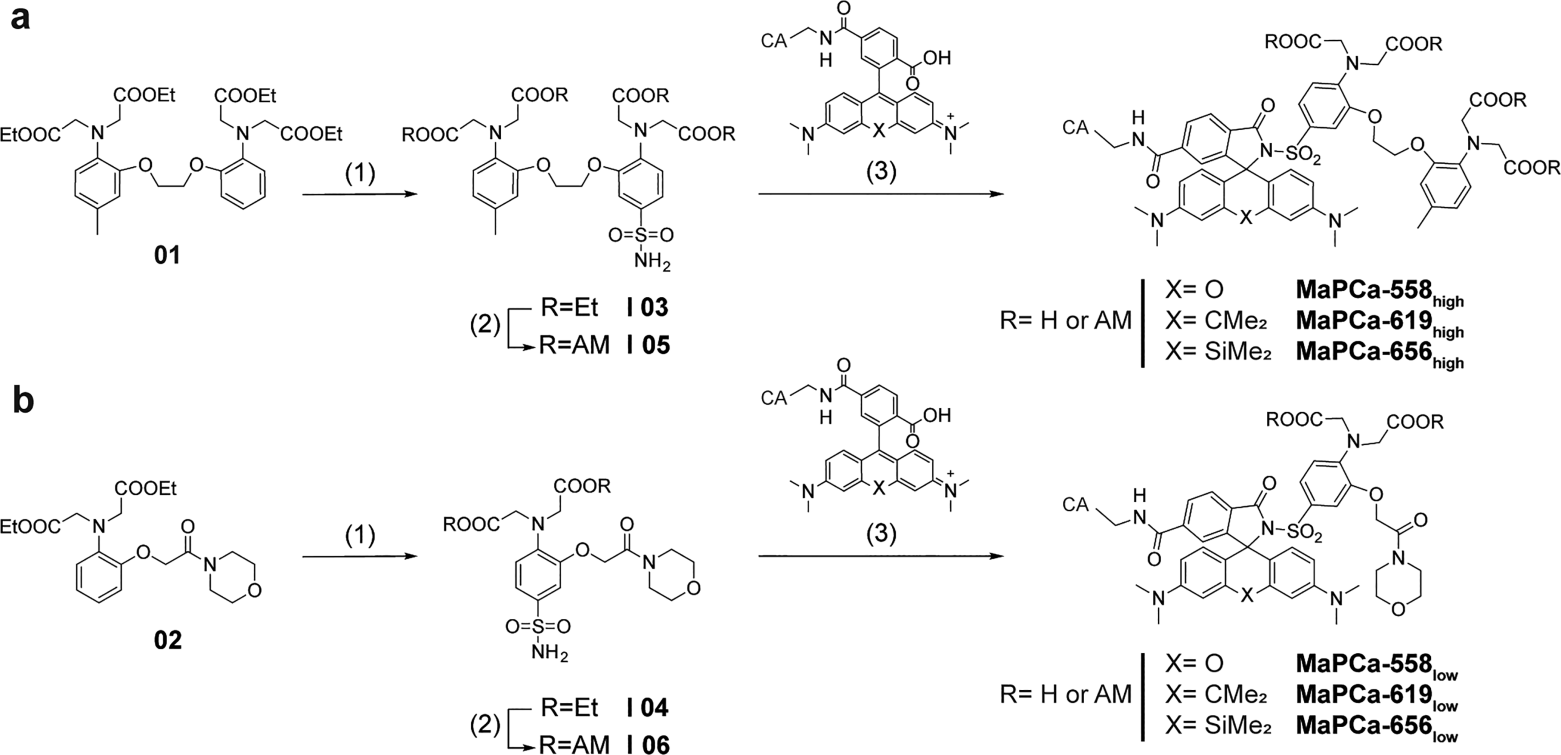
Synthetic pathway for the preparation of MaPCa dyes. The AM esters of the dyes are marked with an additional AM, in contrast to the saponified probe. (a) Synthetic route for MaPCa_high_ (1) (i) HSO_3_Cl, SOCl_2_, CH_2_Cl_2_, 0°C–rt, 24 h and (ii) aq NH_3_, EtOAc, rt, 75%; (2) this step was only performed for the AM probes for the cellular experiments: (i) DMAP, di-*tert*-butyl-dicarbonate, CH_2_Cl_2_, 35 °C, 24 h, (ii) KOH, MeOH/THF, rt, 2 h, (iii) DIPEA, bromomethyl acetate, MeCN, rt, 48 h, and (iv) TFA, CH_2_Cl_2_, rt, 2 h, 61%; (3) (i) fluorophore preactivation with SOCl_2_, pyridine, CH_2_Cl_2_, rt–60 °C, 0.5 h and (ii) DIPEA, DMAP, 60 °C, 1 h, 26–56%; the ethylester was subsequently saponified: KOH, MeOH/THF, rt, 8 h, 42–66%. (b) Synthetic route for MaPCa_low_ (1) (i) HSO_3_Cl, SOCl_2_, CH_2_Cl_2_, 0°C–rt, 24 h and (ii) aq NH_3_, EtOAc, rt, 44%; (2) this step was only performed for the AM probes for the cellular experiments: (i) DMAP, di-*tert*-butyl-dicarbonate, CH_2_Cl_2_/MeCN, 35 °C, 43 h, (ii) KOH, MeOH/THF, rt, 5.5 h, (iii) DIPEA, bromomethyl acetate, MeCN, rt, 21 h, and (iv) TFA, CH_2_Cl_2_, TIPS, rt, 0.5 h, 36%; (3) (i) fluorophore preactivation with SOCl_2_, pyridine, CH_2_Cl_2_, rt–60 °C, 0.5 h and (ii) DIPEA, DMAP, 60 °C, 3.5 h, 13–32%; the ethylester was subsequently saponified: KOH, MeOH/THF, rt, 5 h, 22–58%.

We set out to synthesize a set of such indicators based on the high-affinity calcium chelator BAPTA and the low-affinity chelator MOBHA [2-(2′-morpholino-2′-oxoethoxy)-*N*,*N*-bis(hydroxycarbonylmethyl)aniline]^24^ in combination with commercially available rhodamine–CA substrates TMR–CA, CPY–CA, and SiR–CA, covering the spectrum from 550 to 650 nm (Figure 1b). In a first step, a sulfonamine was attached to the previously described BAPTA–ethylester^25^ (**01**) or MOBHA–ethylester (**02**) *via* chlorosulfonation followed by amination (**03**, **04**). These two intermediates were then coupled to the commercially available rhodamine–CAs TMR–CA, CPY–CA, and SiR–CA using activation by chlorosulfonic acid. The indicators were obtained as free acids after saponification with KOH (Figure 2).

For live-cell experiments, acetoxymethyl (AM) esters of the indicators were synthesized by prior transesterification of the chelator (**05**, **06**) and subsequent coupling to the fluorophore. The AM esters serve to mask the carboxylic acids to ensure cell permeability, but are cleaved inside the cell by endogenous esterases.^26^ We named these indicators MaPCa dyes (for Max Planck calcium sensor), with a postfix expressing the absorption maxima in nm (TMR = 558; CPY = 619; SiR = 656) and the subscripts “*high*” or “*low*” for indicating the calcium affinity range. The AM esters of the dyes are marked with an additional AM, in contrast to the saponified probes. It should be noted that this short and convergent synthetic scheme should enable the conversion of most rhodamine–CAs into calcium sensors in a single step.

### *In Vitro* and *Live-Cell* Evaluation of MaPCa Dyes

**Figure 3:**
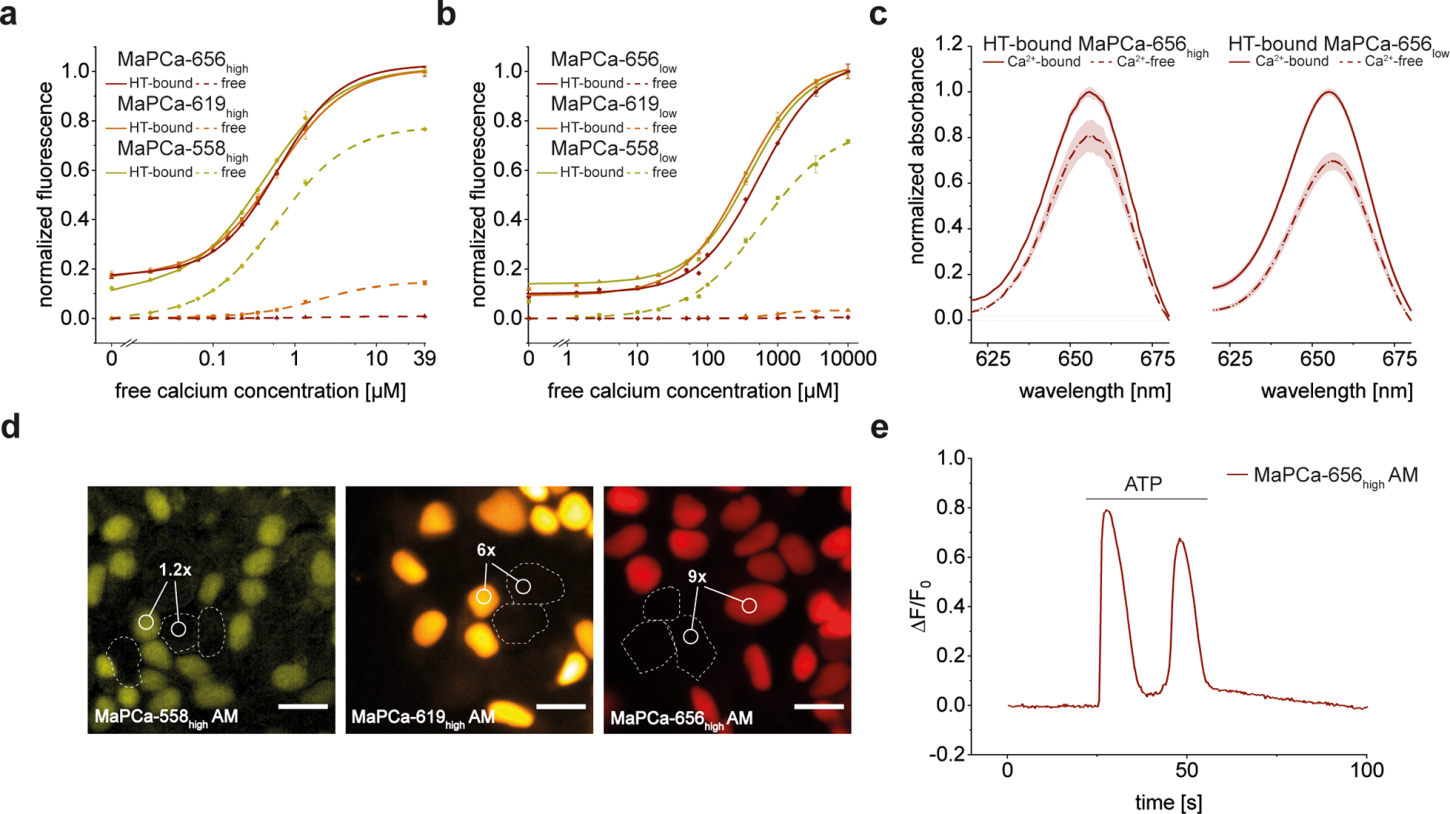
Characterization of MaPCa dyes. (a,b) Calcium titration of (a) MaPCa_high_ and (b) MaPCa_low_. Depicted is the mean (of *n* = 3) with standard deviation. (c) Absorbance spectra of HT-bound MaPCa-656 indicators show calcium-dependent absorbance increase. (d) Fluorescence microscopy images of a co-culture of HaloTag-NLS-expressing and nonexpressing 293 cells. Cells were incubated with 1 μM MaPCa-558_high_ AM (left), MaPCa-619_high_ AM (middle), and MaPCa-656_high_ AM (right) for 2 h and imaged under no-wash conditions. Turn-on numbers represent average of *n* = 200 cells. Scale bar, 20 μm. (e) Exemplary fluorescence trace of 293 stably expressing HaloTag-SNAP-tag fusion proteins in the nucleus, incubated with MaPCa-656_high_ AM and perfused with 100 μM ATP. The occurrence of successive calcium spikes upon ATP perfusion has been described previously.^27^ HT = HaloTag.

The MaPCa dyes’ calcium responsiveness was characterized *in vitro* in the presence and absence of HaloTag measuring their fluorescence intensities at different free calcium concentrations (Figures 3a,b, S3, and S4). As desired, all three high-affinity indicators showed a fluorogenic turn-on upon binding to HaloTag. However, though MaPCa-558_high_ was only slightly fluorogenic (1.3-fold), MaPCa-619_high_ and MaPCa-656_high_ showed a significant 7-fold and even 120-fold increase upon binding to HaloTag, respectively, in the calcium-bound state.

The higher fluorogenicity of MaPCa-656_high_ can be rationalized considering the higher propensity of SiR derivatives to exist in the nonfluorescent spirocyclic form than the corresponding rhodamine and carborhodamine derivatives.^28^ In the calcium-bound state, the dyes possess a high quantum yield of >40% and extinction coefficients of >80,000 M^–1^ cm^–1^, suggesting that they are predominantly in the open state when bound to calcium and HaloTag.^21^ They display calcium affinities in a suitable range for cytosolic measurements [*K*_D_(Ca^2+^): 410–580 nM] with turn-ons of around 6-fold upon calcium binding (Tables 1 and S1). The low-affinity indicators show similar fluorogenicities as the BAPTA variants: the TMR variant (MaPCa-558_low_) shows low fluorogenicity (1.4-fold) upon HaloTag binding, whereas MaPCa-619_low_ (28-fold) and MaPCa-656_low_ (208-fold) are highly fluorogenic. The calcium affinities of these dyes are in the range of 220–460 μM and they show a 7- to 11-fold turn-on upon calcium binding in the calcium-saturated state (Tables 1 and S1). The extinction coefficient of MaPCa-656_low_ is significantly lower than those of the other MaPCa indicators and similar dyes,^21^ suggesting that it is predominantly in the closed state. Nevertheless, its brightness of ∼15 mM^–1^ cm^–1^ is in the same order of magnitude as genetically encoded red-shifted indicators (brightness FR-GECO1c: 9.3 mM^–1^ cm^–1^).^29^ As HaloTag binding reduced the calcium turn-on observed in the free dye (*F*_max_/*F*_0_ = 8–24×; Table S1), we tested if it also affected the calcium-binding kinetics and the selectivity of the indicators against other cations. Stopped-flow measurements of HaloTag-bound MaPCa revealed high *k*_off_ values of above 248 s^–1^, which are significantly higher than those observed for GCaMP6f with 4 s^–1^.^30^ Selectivity measurements revealed a good discrimination against other cations (Figures S5 and S6).

**Table 1:** Photophysical Properties of MaPCa Dyes^a^

	fluorogenicity upon HT7-binding^b^	*F*_max_/*F*_0_ upon Ca^2+^-binding^c^	λ_Ex_/λ_Em_ [nm]	*K*_D_(Ca^2+^) [μM]^c^	brightness [mM^–1^ cm^–1^]^d^
MaPCa-558_high_	1.3	6	558/580	0.41	40
MaPCa-619_high_	7	6	619/632	0.57	55
MaPCa-656_high_	120	6	656/670	0.58	33
MaPCa-558_low_	1.4	7	560/580	224	26
MaPCa-619_low_	28	8	618/633	322	45
MaPCa-656_low_	208	11	655/670	457	15

a HT = HaloTag.

b Fluorescence increase at saturating calcium concentration.

c In HaloTag-bound state.

d At saturating calcium concentration and HaloTag-bound.

We hypothesized that the increase of fluorescence intensity of the MaPCa dyes upon calcium binding should be mainly due to decreased PET quenching. However, MaPCa-656_high_ and MaPCa-656_low_ show a 20–30% increase in absorbance upon calcium binding (Figure 3c). This can be rationalized considering that both indicators, when bound to the HaloTag in the absence of calcium, are not fully in the open state. Calcium binding then weakens the electron-donating effect of the aniline moiety, pushing the equilibrium further to the open, fluorescent state (Figure S7).

For first cellular calcium imaging experiments, AM esters of the MaPCa_high_ indicators were applied to co-cultures of 293 cells stably expressing a nuclear localized HaloTag and 293 cells without HaloTag. Imaging the cells after 2 h of incubation without any washing steps revealed efficient HaloTag labeling (Figures 3d and S8), demonstrating that these molecules are cell permeable. Furthermore, the stable fluorescence signal after 2 h of incubation suggests that AM esters are efficiently hydrolyzed by esterases (Figure S9). The comparison of the cytosolic background fluorescence intensity in nonexpressing cells *versus* the nuclear signal of expressing cells revealed that MaPCa-619_high_ AM and MaPCa-656_high_ AM show excellent signal-to-background ratios (*F*_nuc_/*F*_cyt_ = 6 and 9, respectively) (Figure 3d). This can be rationalized by the high fluorogenicity of these two substrates.

In contrast, the low fluorogenicity of MaPCa-558_high_ AM results in a high background under no-wash conditions (*F*_nuc_/*F*_cyt_ = 1.2) (Figure 3d). As the no-wash protocol can result in prolonged incubation times, we verified that the cell viability of 293 cells is not affected after overnight incubation (Figure S10). Furthermore, all MaPCa_high_ AM indicators translated the calcium concentration increase induced by ATP treatment by a mean fluorescence intensity increase (Δ*F*/*F*_0_) ranging between 0.5 and 2 (Figure 3e). The Δ*F*/*F*_0_ was higher than those we measured with the previously published JF_649_-BAPTA indicator (Figure S11).^15^

### MaPCa Dye Report on Calcium Signaling in Neurons

**Figure 4:**
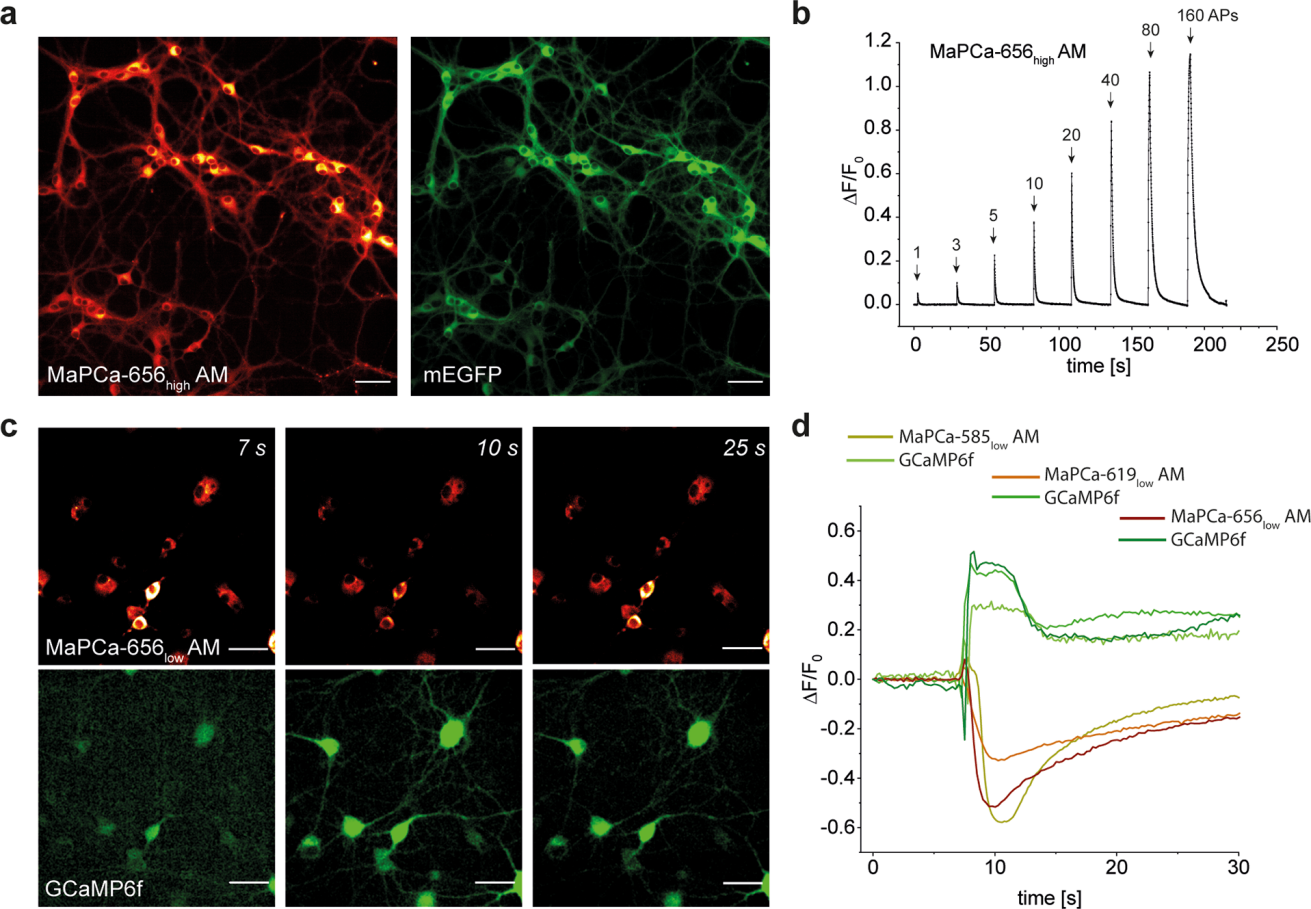
MaPCa dyes can report on calcium flux in primary rat hippocampal neurons. (a) Fluorescence microscopy images of primary rat hippocampal neurons expressing NES-HaloTag-eGFP incubated with 1 μM MaPCa-656_high_ AM and imaged under no-wash conditions; MaPCa-656_high_-channel (left) and eGFP channel (right). Scale bar, 50 μm. (b) Baseline-corrected average trace of stimulated neurons expressing HaloTag and incubated with 1 μM MaPCa-656_high_ AM under no-wash conditions (*n* ≥ 50 cells). APs: 1, 2, 5, 10, 20, 40, 80, and 160. (c) Fluorescence microscopy images of rat hippocampal neurons expressing ER-localized HaloTag and cytosolic GCaMP6f. Cells were incubated with 1 μM MaPCa-656_low_ AM for 2 h and imaged under no-wash conditions. Later, ∼7 s caffeine (final concn: 20 mM) was added. (d) Fluorescence time trace of a representative cell in (c) and of identically treated cells with the indicators MaPCa-558_low_ AM and MaPCa-619_low_ AM (single representative cell) imaged simultaneously with GCaMP6f. Scale bars, 50 μm.

In a next step, the performance of the MaPCa indicator series was evaluated in rat primary hippocampal neurons. For experiments with primary neuronal cultures, the possibility to perform the labeling without any washing steps is important, as such steps are known to disturb viability of primary cell cultures.^31^ rAAV transduced rat primary hippocampal neurons expressing HaloTag–mEGFP strictly in the cytoplasm were labeled with either MaPCa-619_high_ AM or MaPCa-656_high_ AM and imaged under no-wash conditions. Both dyes led to efficient and homogeneous HaloTag labeling without the occurrence of a significant background signal or unspecific staining. The comparison with JF_649_ BAPTA AM revealed a significantly improved signal-to-background ratio for MaPCa-656_high_.^15^ In contrast, MaPCa-558_high_ AM required a washing step to reach the results similar to MaPCa-619_high_ AM and MaPCa-656_high_ AM (Figures 4a and S12). To test the sensitivity of the high-affinity MaPCa indicators, labeled neurons were stimulated with a distinct number of action potentials (APs) using electric field stimulation.^32^ All dyes allowed the detection of a single AP with Δ*F*/*F*_0_ values ranging between 3% (MaPCa-558_high_ AM) and 6% (MaPCa-656_high_ AM), whereas Δ*F*/*F*_0_ of 120% was obtained using MaPCa-656_high_ AM with a 160 AP burst, a visible improvement to the 60% we obtained with JF_649_-BAPTA AM (Figures 4b and S13, Video S1). The lower calcium affinity of the MaPCa_low_ series allows reporting of calcium fluctuations in compartments with high basal calcium concentrations such as the ER (Ca^2+^ concn: ∼500 μM).^19^ Therefore, the MaPCa dyes were targeted to the ER through rat hippocampal neuron transduction localizing a HaloTag-SNAP-tag fusion in the ER. Co-staining of SNAP-tag or utilization of an ER tracker confirmed efficient and specific labeling of HaloTag with MaPCa_low_ dyes under no-wash conditions, with the exception of MaPCa-558_low_ AM that required a washing step to reduce the background (Figures S14 and S15). The ER is a calcium store which, upon stimulation, can release calcium into the cytosol. Here, the RyR2 channel plays a crucial role as a calcium-induced calcium release channel.^33^ As the red-shifted wavelengths of the MaPCa dyes do not spectrally overlap with the GFP channel, we multiplexed the MaPCa signal from the ER with a cytosolic GCaMP6f, that is, to simultaneously image calcium efflux from the ER and cytosolic influx upon stimulation. Specifically, rat hippocampal neurons were double transduced using rAAVs expressing both constructs individually and then labeled with the MaPCa_low_ AM indicators. Upon addition of caffeine, a RyR2 stimulant,^33,34^ we could simultaneously record a signal decrease in the ER due to calcium efflux (MaPCa_low_ AM) and a concomitant signal increase in the cytosol due to calcium influx (GCaMP6f) (Figures 4c,d and S16, Video S2). This demonstrates how MaPCa AM dyes allow, in combination with established GCaMP sensors, visualization of the complex interplay between calcium pools in different cellular compartments in a time-resolved manner.

### Bioluminescence as a Readout

**Figure 5:**
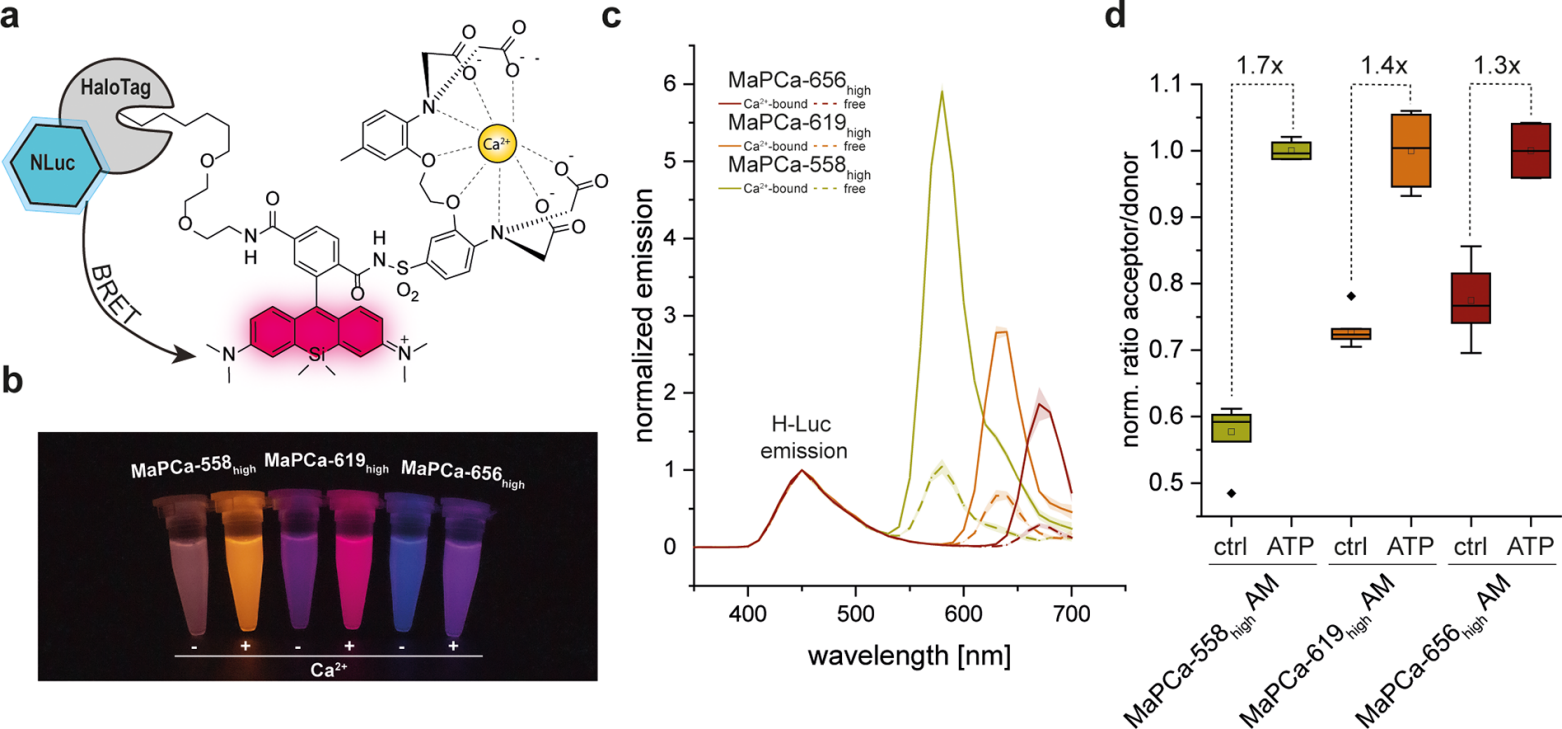
Characterization of MaPCa_high_-based bioluminescent indicators. (a) Bioluminescent H-Luc transfers energy (BRET) to bound MaPCa dyes. (b) Picture of Eppendorf tubes filled with H-Luc labeled MaPCa_high_ dyes in the absence or presence of calcium. (c) Normalized *in vitro* emission spectra of H-Luc labeled MaPCa_high_ dyes, with and without calcium. (d) Normalized acceptor–donor ratio of 293 cells expressing H-Luc in the nucleus and labeled with 1 μM MaPCa_high_ AM dyes. Shown is the ratio of control wells and wells treated with 100 μM ATP and 5 μM thapsigargin. Maximum incubation time is 2 min. *n* ≥ 4 wells per condition. The boxes represent the interquartile range between 25th and 75th percentile whereas the vertical line represents the 5th and 95th percentile. The horizontal line depicts the median and the empty square depicts the mean value. Outliers are represented as points.

The MaPCa dyes could potentially also be used for the labeling of H-Luc, a chimera between HaloTag and the furimazine-dependent luciferase NanoLuc.^35^ Labeling of H-Luc with rhodamine dyes can result in efficient BRET from NanoLuc to the bound rhodamine, such that emission at both 450 nm and at the emission wavelength of the bound rhodamine can be observed. We hypothesized that labeling H-Luc with MaPCa dyes would lead to the development of bioluminescent calcium indicators with tunable emission wavelengths with up to far-red light emission (Figure 5a). Existing bioluminescent calcium indicators, such as orange CAMBI,^36^ GLICO,^37^ LUCI-GECO1,^38^ CeNL,^39^ or CalfluxVTN^40^ rely exclusively on fluorescent proteins that possess emission maxima restricted below 600 nm. We therefore labeled H-Luc with the MaPCa dyes and recorded the emitted light upon addition of furimazine in the absence and presence of calcium. As is already apparent by eye (Figure 5b), the color of the emitted light dramatically depends on both, the presence of calcium as well as the nature of the MaPCa dye attached to H-Luc (Figure 5c). The efficiency of BRET is largest for MaPCa-558_high_ attached to H-Luc, as it has the largest spectral overlap with the BRET donor. As the intensity of the emission of the MaPCa dye depends on the concentration of calcium, measuring the ratio of the intensity of emitted light at 450 nm *versus* the intensity of the light emitted at the emission maximum of the rhodamine dye can thus be used to record changes in calcium concentrations (Figures 5b,c and S17). The maximal change in ratio ranged from 6.5 for H-Luc labeled with MaPCa-656_high_ to 4.2 for H-Luc labeled with MaPCa-619_high_. The H-Luc-MaPCa ratio changes are comparable to those of previously described ratiometric, bioluminescent calcium sensors, and to the best of our knowledge, H-Luc labeled with MaPCa-656_high_ is the first bioluminescent calcium indicator with emission in the far red. To demonstrate how these ratiometric bioluminescent calcium sensors can be exploited for cellular applications, Flp-In 293 cells with a nuclear H-Luc expression were labeled with the MaPCa_high_ AM dye series. The cells were then exposed to a solution of ATP and thapsigargin and the emission ratio of the emitted light was recorded. A significant change in luminescence emission ratio for all three MaPCa dyes was observed upon drug treatment, the value being the highest for MaPCa-558_high_ AM (1.7-fold) and the smallest for MaPCa-656_high_ AM (1.3-fold) (Figure 5d), whereas no change in luminescence intensity could be observed in the absence of the dye (Figure S18). Each channel’s luminescence intensity was integrated in less than 500 ms, allowing changes in calcium concentrations to be followed with good temporal resolution. The *z*-factor is a measure for the statistical effect size used to judge the suitability of an assay for high-throughput screening (HTS) approaches. Flp-In 293 cells expressing H-Luc labeled with MaPCa-558_high_ AM presented a *z*-value of 0.58 upon ATP/thapsigargin treatment, highlighting the suitability of such bioassays for HTS (*z*-factors ≥ 0.5 indicate excellent suitability).^41^ It should be noted that the ratiometric readout of the BRET sensor in principle could also be exploited for the determination of absolute Ca^2+^ concentration. However, the dependency of such analyses on the labeling efficiencies in our opinion would make such experiments impractical (Figure S19). Finally, low-affinity bioluminescent calcium indicators could be generated by labeling H-Luc with the MaPCa_low_ indicators, demonstrating the modularity of the approach (Figure S20).

## Conclusions

We have introduced a new design principle for the development of localizable and fluorogenic calcium indicators. Using this strategy, we have developed several indicators with different colors, up to the far-red, and with different calcium affinities. What distinguishes these indicators from previous work is the good permeability of the probes and the possibility to use them without additional washing steps to remove the unbound indicator. This greatly facilitates their use in most biological applications. Furthermore, they are accessible through a short and modular synthetic pathway. We demonstrated applications of the indicators in rat hippocampal neurons, where the high-affinity indicator MaPCa_high_ could detect single APs under no-wash conditions. The low-affinity indicator MaPCa_low_ was successfully localized in the ER, where it could detect calcium efflux isochronal to increase in cytosolic calcium detected by GCaMP6f. We furthermore developed the first far-red bioluminescent calcium indicator by coupling MaPCa with H-Luc, a bioluminescent HaloTag. The use of H-Luc-MaPCa in cells also demonstrated the possibility to use such bioassays in HTS approaches. These examples underscore the versatility of these calcium indicators and their ease of use.

Finally, the established design principles of these calcium indicators should be transferable to metal ions other than calcium.^42,43^

## Supporting Information Available

Synthetic pathways, titration curves, absorption/emission spectra, quenching mechanism, fluorescence and transmitted light microscopy images, widefield fluorescence microscopy data and images, *in vitro* tests, cell culture, microscopy, synthesis and characterization, NMR spectra, photophysical properties of dyes, list of plasmids and stable cell lines, titers of applied rAAVs, cloning, protein expression and purification data, optical spectroscopy data, bioluminescence measurements, other live-cell experiment details, and protein sequences (PDF)
Primary hippocampal neurons expressing HaloTag in the cytosol labeled with MaPCa-565high AM and stimulated using electric-field stimulation (corresponding trace Figure 4b) (AVI)
Primary hippocampal neurons expressing HaloTag in the ER labeled with MaPCa-656low AM and stimulated using caffeine (corresponding trace Figure 4d) (AVI)

## Supplementary Material

ja2c01465_si_001.pdf

ja2c01465_si_002.avi

ja2c01465_si_003.avi
